# A ng/L Level LC-MS Method Using Membrane SPE as Sampling Technology: Determination of Nine Halobenzoquinones in Potable Water

**DOI:** 10.3390/molecules29122856

**Published:** 2024-06-15

**Authors:** Qin Huang, Hua Zhou, Xianglun Wu, Jiaqi Jiang, Bingdong Zhan, Pinggu Wu

**Affiliations:** 1 Quzhou Center for Disease Control and Prevention, Quzhou 324000, China; hq6045@foxmail.com (Q.H.); wuxianglun2024@foxmail.com (X.W.); jiangjiaqi1118@foxmail.com (J.J.); bd_zhan@126.com (B.Z.); 2 Lab of Physicochemical Research, Department of Physicochemical & Toxicology, Zhejiang Provincial Centre for Disease Control and Prevention, Hangzhou 310051, China; pgwu@cdc.zj.cn

**Keywords:** membrane solid-phase extraction (MSPE), halobenzoquinones (HBQs), liquid chromatography–mass spectrometry (LC-MS), potable water

## Abstract

A promising method was established for the determination of nine halobenzoquinones (HBQs) in potable water by membrane solid-phase extraction (MSPE) pretreatment and the liquid chromatography–mass spectrometry (LC-MS) method. A 500 mL water sample was taken for enrichment by the SDB-RPS membrane, which was previously activated by methanol and ultrapure water. The sample was eluted with methanol and re-dissolved with the initial mobile phase after nitrogen blowing. Then, it was detected in negative ion mode using the working curve, and HBQs were quantified by the external standard method. The linearity was satisfactory in the concentration range of 4-1000 ng/L, with correlation coefficients of 0.9963~0.9994. The recoveries were 73.5~126.6% at three spiked levels, with relative standard deviations (RSDs) of 6.8~15.5%. The limits of detection (LOD, S/N = 3) values were 0.1~0.7 ng/L. The results demonstrate that the MSPE-LC-MS method is reliable, rapid, and sensitive for the simultaneous analysis of nine HBPs in potable water.

## 1. Introduction

As a critical public health measure, disinfection can effectively kill bacteria and viruses in potable water and prevent intestinal and other infectious diseases [1]. Common disinfection methods include ultraviolet disinfection [2], chlorination [3] and ozone disinfection [4], with chlorination being the most common. However, in the disinfection process, the disinfectant chemically reacts with some natural organic substances in the raw water, as well as chlorine or bromine, resulting in a series of disinfection by-products (DBPs) [5,6,7]. Epidemiological investigations have found that DBPs may increase the risk of bladder cancer and some other health problems in humans [8,9]. Nevertheless, epidemiological risk assessment of regulated DBPs (e.g., trihalomethanes, haloacetic acid, etc.) does not yet explain the increasing risk of bladder cancer [10], suggesting that some uncontrolled DBPs at lower levels in potable water may be a bigger potential hazard. Halobenzoquinones (HBQs) have emerged as a DBP detected in potable water in recent years [11,12]. Toxicological studies have shown that HBQs are more cytotoxic and genotoxic than regulated DBPs [13,14]. HBQs can also induce the production of intracellular reactive oxygen species, which reduces the antioxidant enzyme activity of cells, leading to oxidative DNA damage [15,16,17]. Given that HBQs may pose a greater health risk to the population and that matching standard detection methods are still lacking in China, it is vital to establish highly sensitive monitoring technology to fill this gap.

As the content of HBQs in potable water is at a nanogram level [18], the sensitivity of gas chromatography (GC) [19] and high-performance liquid chromatography (HPLC) [20] makes it difficult for these techniques to meet the actual requirements. The advantages of high sensitivity, accuracy, and specificity make liquid chromatography–mass spectrometry (LC-MS) [21,22,23] appropriate for the qualitative and quantitative determination of contaminants in water samples. The pretreatment of HBQs in water is mainly based on solid-phase extraction (SPE), which has a simple operation and low solvent consumption [24]. Unfortunately, the traditional SPE column is only suitable for small-volume sampling, as it is limited by its surface area and column capacity. It takes longer enrichment time (the flow rate is generally 10–15 mL/min), which may lead to clogging when processing samples containing particles [25]. The large surface area of membrane solid-phase extraction (MSPE) provides a homogeneous and dense extraction bed to accelerate the extraction efficiency (the flow rate can reach approximately 50–100 mL/min) [26,27]. Also, the MSPE method is not easily blocked, which makes it especially suitable for the rapid enrichment of large-volume water samples with more impurities [28,29].

In this paper, a promising method based on MSPE enrichment and LC-MS confirmation for analyzing HBQs in potable water is proposed. This method was multi-conditionally optimized and methodologically validated for the identification and quantification of nine HBQs in drinking water.

## 2. Results and Discussions

### 2.1. Optimization Conditions

#### 2.1.1. Optimization of MS Conditions

**Figure 1:**
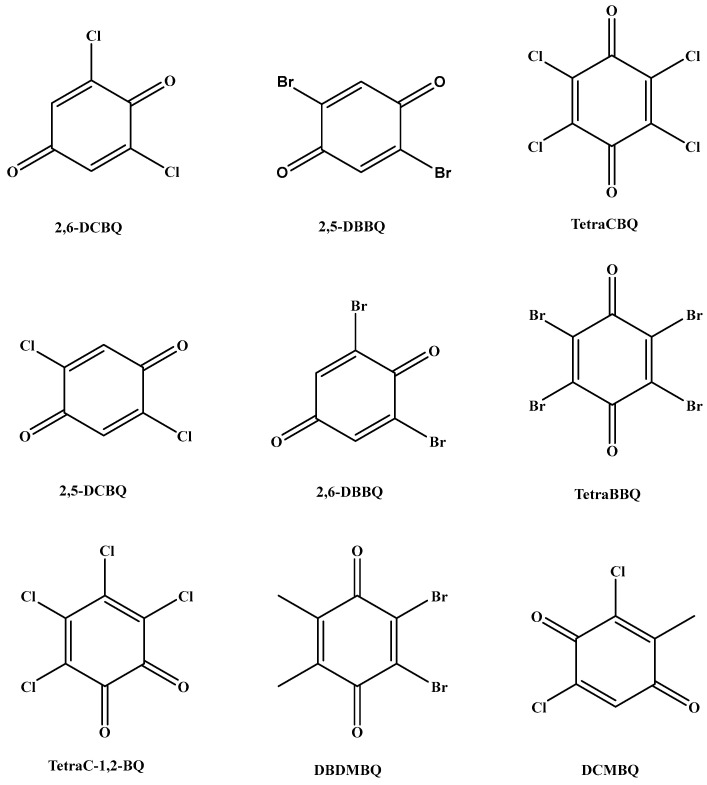
The chemical structures of nine selected analytes in this work.

A 50 μg/L mixed standard solution diluted with methanol–water (1:1, *v*/*v*, containing 0.25% formic acid) was injected into the instrument at a flow rate of 7 μL/min by a peristaltic pump. The optimization of MS conditions was carried out in the electrospray negative ion (ESI−) mode in order to select suitable parent ions. In the ESI- mode, a primary mass spectrometry scan was first performed to determine its quasi-molecular ion peak to select the suitable stable precursor ions (Q1). Then, the precursor ions were collisionally dissociated to obtain the fragment ion information of the target compounds by secondary mass spectrometry ion scanning to select the product ions (Q3). Two product ions with stronger response and less interference were selected as the qualitative and quantitative ions for the optimization of collision energy (CE) and declustering voltage (DP). To obtain better quantitative reproducibility, the sampling points of the chromatographic peaks were obtained in the range of 15~20 by setting the dwell time. 2,5-DCBQ and 2,6-DCBQ, 2,5-DBBQ and 2,6-DBBQ, and TetraCBQ and TetraC-1,2-BQ are three pairs of tautomers with the same parent and daughter ions. Figure 1 shows the chemical structures of nine selected analytes in this work. Table 1 lists the detailed MS parameters of nine HBQs.

#### 2.1.2. Optimization of Chromatographic Conditions

**Figure 2:**
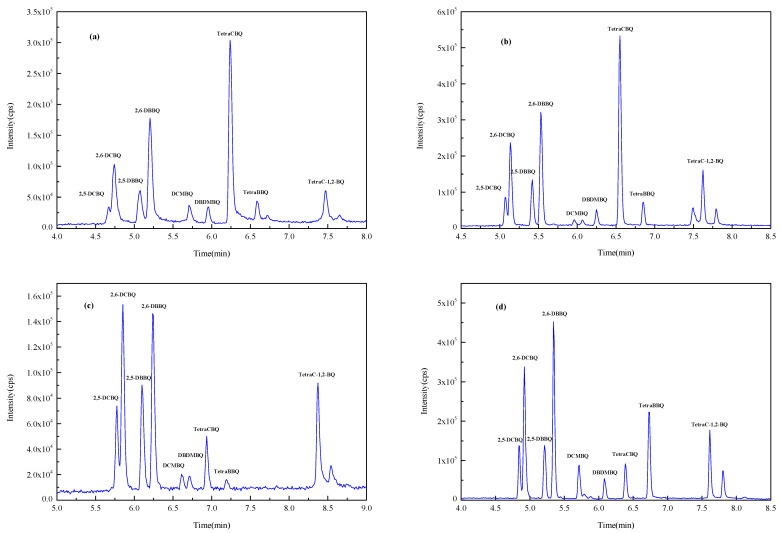
The TIC spectrum of nine HBQs (separated by four columns). (**a**) ACQUITY UPLC BEH C18 column (1.7 µm, 2.0 mm × 100 mm); (**b**) ACQUITY UPLC HSS T3 column (1.8 µm, 2.1 mm × 100 mm); (**c**) ACQUITY UPLC BEH C18 column (1.7 µm, 3.0 mm × 100 mm); (**d**) Acclaim TM RSLC 120 C18 column (2.2 µm, 2.1 mm × 100 mm).

In this experiment, Waters ACQUITY UPLC BEH C18 (1.7 µm, 2.0 mm × 100 mm) (Milford, CT, USA), Waters ACQUITY UPLC BEH C18 (1.7 µm, 3.0 mm × 100 mm) (Milford, CT, USA), Waters ACQUITY UPLC HSS T3 (1.8 µm, 2.1 mm × 100 mm) (Milford, CT, USA), and Thermo Acclaim TM RSLC120 C18 (2.2 µm, 2.1 mm × 100 mm) (Waltham, MA, USA) columns with methanol–water as the mobile phase were investigated for the gradient elution optimization experiments. Figure 2 shows the total ion chromatogram (TIC) spectrum of nine HBQs using four columns. It was found that 2,5-DCBQ and 2,6-DCBQ were difficult to effectively separate when using the ACQUITY UPLC BEH C18 column (1.7 µm, 2.1 mm × 100 mm) and the ACQUITY UPLC HSS T3 column, while the ACQUITY UPLC BEH C18 (1.7 µm, 3.0 mm × 100 mm) column and the Acclaim TM RSLC120 C18 column were effective in separating the DCBQ isomers. The peak sensitivity of DCMBQ, DBDMBQ, and TetraBBQ was not satisfactory using the ACQUITY UPLC BEH C18 (1.7 µm, 3.0 mm × 100 mm) column for separation. The Acclaim TM RSLC 120 C18 column achieved baseline separation of the DCBQ isomers with better peak shape and resolution. According to the literature, the Acclaim TM RSLC 120 C18 column with methanol as solvent B can obtain good separation efficiency for the majority of analytes [30,31]. Therefore, the Acclaim TM RSLC 120 C18 column was selected as the analytical column.

The mobile phase was evaluated in the experiment. The same elution procedure and chromatographic conditions were used in all experiments. Water was used for the aqueous phase, and acetonitrile and methanol were used for the organic phase. When optimized with acetonitrile as the organic phase in the gradient procedure, it could not achieve effective separation of 2,5-DCBQ and 2,6-DCBQ. This might be due to its strong elution capacity [32]. Meanwhile, 2,5-DCBQ and 2,6-DCBQ could be separated better when methanol was used as the organic phase.

In addition, the ionic strength and pH value of the mobile phase affected the response of the targets. By comparing the addition of 0.1% FA, 0.05% ammonia, and 2 mmol/L ammonium acetate to the aqueous phase, the peak shape and sensitivity of the target peaks were assessed. The results showed that the addition of ammonia and ammonium acetate to the mobile phase decreased the response of the target peak, whereas the addition of FA obviously improved the sensitivity. Previous literature has elaborated that the major molecular ions of HBQs correspond to [M + H^+^ + 2e^−^], which is equivalent to [M + H]^−^ and the radical M^−•^ [22]. The addition of FA promoted the formation of the parent ion [M + H]^−^ in the negative ion mode, consequently increasing the ionization efficiency and improving detection sensitivity. By increasing the concentration of FA and optimizing the parameters of the gradient elution procedure, the injection volume, and the flow rate, 0.2% FA as the aqueous phase and methanol as the organic phase were finally determined, and excellent separation and sensitivity of the target peaks were obtained.

It was found that the temperature of the column could affect the sensitivity of the analytes being measured. On the basis of the above optimal conditions, the separation and sensitivity of the analytes were compared at column temperatures of 35, 40, 45, and 50 °C. The separation was excellent, and the sensitivity was the highest at a column temperature of 45 °C. Therefore, 45 °C was chosen for the chromatographic analysis.

The component of the reconstituted solution affected the sensitivity and the peak shape as well. When the proportion of methanol was higher than 20%, the peak shape of the targets performed poorly, and the sensitivity was low. We found that 5% methanol in water (*v*/*v*) could obtain a fine peak shape. The sensitivity was significantly improved as FA was added to the reconstituted solution. Therefore, sensitivity values of 0.05%, 0.1%, 0.2%, and 0.3% FA were compared. The results showed that 0.2% FA achieved the highest response due to the higher concentration of FA, which reduced the response. Therefore, the reconstituted solution used in our experiment was 5% methanol in water (*v*/*v*) containing 0.2% FA.

### 2.2. Selection of SPE Membrane

**Figure 3:**
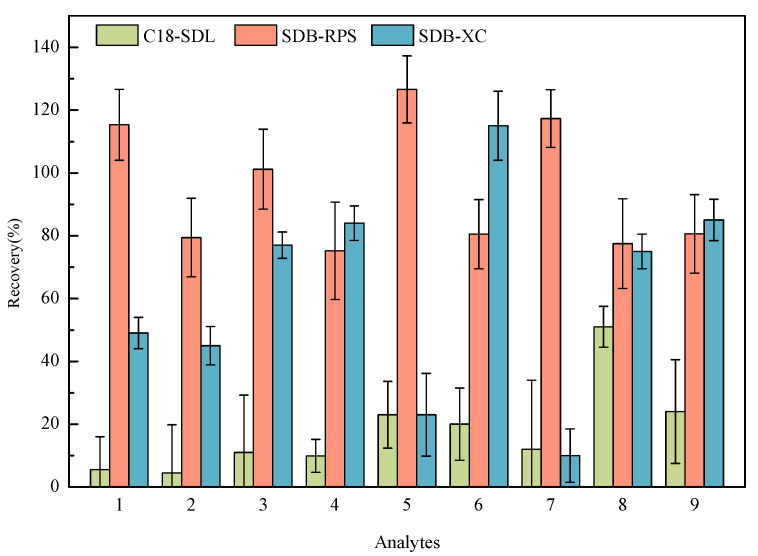
Effect of three SPE membranes on the recoveries of nine analytes (1: 2,5-DCBQ; 2: 2,5-DBBQ; 3: 2,6-DCBQ; 4: 2,6-DBBQ; 5: TetraCBQ; 6: TetraBBQ; 7: TetraC-1,2-BQ; 8: DBDMBQ; 9: DCMBQ).

Three kinds of MSPE materials (C18-SDL, SDB-XC, and SDB-RPS) were used to extract the samples with a spiked concentration of 20 ng/L. Figure 3 presents the recoveries of the nine HBQs using three MSPE materials for pretreatment. For C18-SDL, the recoveries for most targets were less than 30%. When pretreated with SDB-XC, it was slightly lower than that with SDB-RPS. SDB-RPS exhibited such good separation properties that the recoveries reached 80–120% for nine analytes. SDB-RPS is a styrene–diethylene–benzene-based resin, which is a spherical, porous, and cross-linked copolymer resin particle [33]. It is more hydrophilic and contributes to the modified reversed-phase sulfonic acid group, which is capable of extracting a wide range of organic analytes simultaneously [34,35]. Considering these factors, the SDB-RPS membrane was chosen for the adsorption of the analytes being measured.

### 2.3. Selection of Eluent

The elution effects were conducted by comparing 10 mL of dichloromethane, 10 mL of methanol, 10 mL of ethyl acetate, and 10 mL of methanol containing 0.25% formic acid. At the same condition, the recovery of methanol as the eluent was much higher than that of dichloromethane and ethyl acetate. Because the content of formic acid has a large effect on the substance to be measured and the precision of the method can also be affected by the concentration change of formic acid after elution and nitrogen blowing, methanol was chosen as the eluent.

The elution experiments were carried out using 9 mL of methanol three times (3 mL each time). More than 95% of the targets could be eluted for the first time. In order to induce the nitrogen blowing time and also consider the elution effect, the volume of eluent chosen was 4 mL.

### 2.4. Method Validation

**Figure 4:**
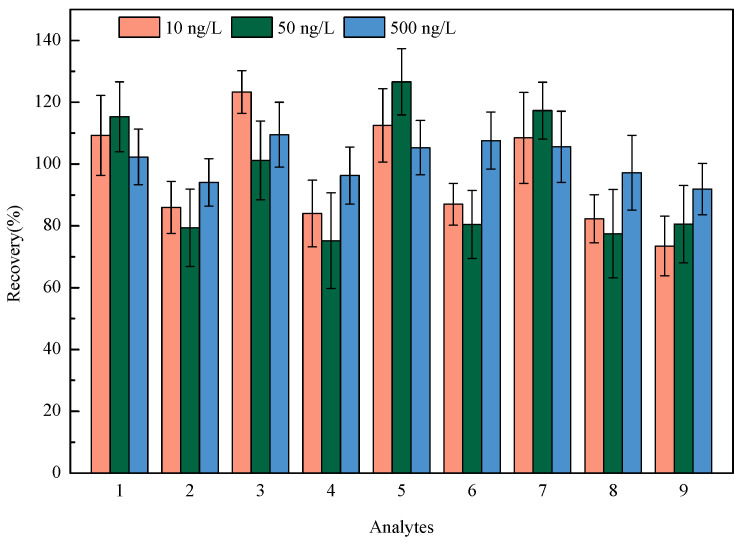
Recoveries of nine HBQs at three spiked levels (1: 2,5-DCBQ; 2: 2,5-DBBQ; 3: 2,6-DCBQ; 4: 2,6-DBBQ; 5: TetraCBQ; 6: TetraBBQ; 7: TetraC-1,2-BQ; 8: DBDMBQ; 9: DCMBQ).

The linearity of nine HBQs was measured in the range of 4-1000 ng/L, with correlation coefficients (R^2^) of 0.9963~0.9994. The negative tap water was marked as the blank matrix for recovery and precision experiments. The recovery and precision of the method were operated by spiking standard solutions at three levels (10, 50, and 500 ng/L) and measured in parallel intra-day (*n* = 6). The recoveries ranged from 73.5% to 126.6%, with the relative standard deviations (RSDs) ranging from 6.8% to 15.5% (see Figure 4). The limits of detection (LODs) of the proposed method for the analysis of nine HBQs were 0.1–0.7 ng/L, which is estimated as the concentration when the signal-to-noise ratio equals 3. Also, the limits of quantification (LOQs) of the proposed method for the analysis of nine HBQs were 0.3–2.1 ng/L, which is estimated as the concentration when the signal-to-noise ratio equals 10. The detailed methodology parameters are listed in Table 2.

### 2.5. Practical Application

**Figure 5:**
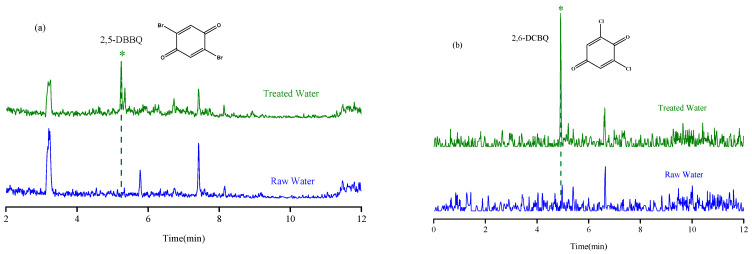
The chromatograms of raw water and treated water with analytes detected. (**a**) 2,5-DBBQ; (**b**) 2,6-DCBQ.

The established MSPE-LC-MS method was applied for the determination of nine HBQs in potable water in Quzhou City. Both source water and disinfected drinking water were investigated. A total of 14 water samples (including 1 source water, 2 finished water, 7 tap water, 2 bottled water, and 2 well water) were collected. The HBQs in potable water were identified on the basis of matching the retention time and MRM transitions with those of the standards. The results showed that raw, bottled water, and well water were not detected; 2,5-DBBQ was detected in two finished water (2.4–3.0 ng/L) and seven tap water (2.3–5.1 ng/L) samples; 2,6-DCBQ (2.0 ng/L) was detected in one tap water sample; and the other HBQs were not detected. The chromatograms comparing raw water and treated water are shown in Figure 5**.** Moreover, the established method was compared with other methods in the literature for the determination of HBQs (Table 3). From the comparison, it is obvious that the method established herein saves time, saves the use of organic solvents, has a wider linear range, and has a higher sensitivity. In addition, this method eliminates additional steps such as liquid–liquid extraction, greatly improving the efficiency of analysis and reducing the workload of laboratory staff.

## 3. Materials and Methods

### 3.1. Materials and Reagents

Methanol and acetonitrile of chromatographic grade were purchased from Merck (Darmstadt, Germany). Ammonia and formic acid (FA) of chromatographic grade were purchased from Aladdin (Shanghai, China). Ammonium acetate of chromatographic grade was purchased from Anaqua Chemicals Supply (Wilmington, NC, USA). The solid-phase extraction membranes (diameter 25 mm) adopted in this research (C18-SDL, SDB-XC, and SDB-RPS) were purchased from CDS Analytical (Oxford, NC, USA). 2,5-Dichloro-1,4-benzoquinone (2,5-DCBQ, 99.3%), 2,6-dichloro-1,4-benzoquinone (2,6-DCBQ, 99.5%), 2,5-dibromo-1,4-benzoquinone (2,5-DBBQ, 98.1%), tetrachloro-1,4-benzoquinone (TetraCBQ, 99.4%), 3,4,5,6-tetrachloro-1,2-benzoquinone (TetraC-1,2-BQ, 99.7%), and 2,3,5,6-tetrabromo-1,4-benzoquinone (TetraBBQ, 99.9%) were purchased from Tanmo Co., Ltd. (Beijing, China). 2,6-Dibrosmo-1,4-benzoquinone (2,6-DBBQ, 10 µg/mL), 2,6-dichloro-3-methyl-1,4-benzoquinone (DCMBQ, 10 µg/mL), and 2,3-dibromo-5,6-dimethyl-1,4-benzoquinone (DBDMBQ, 99.9%) were purchased from Alta Scenitific Co., Ltd. (Tianjin, China). Ultrapure water was prepared by the Milli-Q-plus water purification system. The raw and treated water samples were collected from a local water treatment plant using chlorination as a disinfection method. All chemical reagents used in this work were used directly without further purification.

### 3.2. Instruments and Equipment

A Shimadzu 30AD liquid chromatography (LC) (Shimane,, Japan)coupled to an AB SCIEX 5500Qtrap mass spectrometer (MS) (Boston, MA, USA) equipped with a Thermo Fisher Acclaim TM RSLC 120 C18 column (2.2 µm, 2.1 × 100 mm)(Milford, MA, USA) was used to identify and quantify the nine target analytes. An IKA vortex mixer (Staufen, Germany) and a Beckman high-speed refrigerated centrifuge (California, CA, USA) were used during extraction and purification.

### 3.3. LC-MS Analysis

The chromatographic separation was performed on an Acclaim TM RSLC 120 C18 column (2.2 µm, 2.1 × 100 mm) with a flow rate of 0.4 mL/min. The operating column temperature was 45 °C, and the injection volume was 10 μL. The mobile phase consisted of 0.2% FA (*v*/*v*) in water (eluent A) and methanol (eluent B). The gradient elution procedure was as follows: 0 → 1 min, 5%B; 1 → 8 min, 5% → 100% B; 8 → 9 min, 100%B; 9 → 10 min, 100% → 5%B; 10 → 11 min, 5%B.

Mass spectrometry analysis was performed using negative electrospray ionization (ESI-) in the multiple reaction monitoring (MRM) scanning mode. The ion source temperature was set to 500 °C. The corona needle current and the ion voltage were −3.0 mA and −4 500 V, respectively. Nitrogen was used in all cases. Gas operating conditions were as follows: curtain gas (CUR) pressure: 40 psi; nebulizer gas (gas1): 55 psi; heating gas (gas2): 55 psi; collision gas: high.

### 3.4. Preparation of a Standard Working Curve

A certain amount of solid standard was weighed into a 1.0 mL volumetric flask, dissolved with methanol, and fixed to the scale to obtain a concentration of 1000 mg/L of standard stock solution. Then, it was diluted to 10 mg/L with methanol to acquire the intermediate solution. Then, 100 μL of a single intermediate solution was transferred to a 1.0 mL volumetric flask and fixed with methanol to the scale to obtain a mixed standard solution with a concentration of 1000 μg/L. Next, 500 mL of ultrapure water was taken to prepare a series of standard working solutions of 4, 10, 20, 40, 100, 200, and 1000 ng/L, which were used for the working curve.

### 3.5. Sample Pretreatment

The water samples were collected in 4 L brown glass bottles and acidified with 0.25% FA (*v*/*v*, pH 2.6–2.8) immediately after collection to quench free chlorine and stabilize the target analytes. The samples were in coolers with ice packs when transported to the laboratory and were analyzed within 24 h. In contrast, a travel blank sample and an MSPE blank sample (1 L of optima water, acidified with 0.25% FA) were also analyzed in each batch. Prior to pretreatment, the SDB-RPS membrane was activated by 5 mL of methanol and 5 mL of ultrapure water. Next, 500 mL of water samples were taken into the activated membrane and enriched with a flow rate of 50 mL/min through the extraction membrane. After enrichment, the sample was eluted with 5 mL of ultrapure water and vacuumed for 5 min. Then, the sample was eluted with 4 mL of methanol for nitrogen and blown to near dryness. Finally, it was reconstituted with methanol in water (5:95, *v*/*v*) containing 0.25% FA to a final volume of 1.0 mL and passed through a 0.22 µm filter membrane for further determination.

## 4. Conclusions

In this paper, the SDB-RPS membrane was selected as the MSPE material to adsorb nine HBQs, and the HPLC-MS technique was established for the determination of nine HBQs in potable water. The chromatographic columns, reconstituted solution, column temperature, SPE membrane, and elution effects were assessed to determine the optimal condition. Satisfactory linearity and recoveries were acquired, which were methodologically reliable. The established method is reliable, fast, sensitive, and accurate, and it can be applied for the determination of nine HBQs in potable water. The proposed method provides a new choice for simultaneously monitoring HBQs in water.

## Figures and Tables

**Table 1:** Mass parameters of nine HBQs.

Analyte	ESI (−/+)	Retention Time (min)	Q1 (*m*/*z*)	Q3 (*m*/*z*)	DP (V)	CE (eV)
2,5-DCBQ	−	4.86	176.9	112.9 */34.9	−90	−23/−30
2,5-DBBQ	−	5.22	266.8	78.8 */80.9	−105	−50/−50
2,6-DCBQ	−	4.93	176.9	112.9 */34.9	−90	−23/−30
2,6-DBBQ	−	5.36	266.8	78.8 */80.9	−105	−50/−50
TetraCBQ	−	6.39	245.0	34.9 */208.8	−124	−44/−20
TetraBBQ	−	6.74	424.8	78.9 */81.0	−110	−70/−70
TetraC-1,2-BQ	−	7.62	245.0	34.9 */208.8	−124	−44/−20
DBDMBQ	−	6.09	309.0	214.8 */142.8	−130	−21/−34
DCMBQ	−	5.72	191.0	35.0 */127.0	−50	−35/−22

* indicates the quantitative transition.

**Table 2:** Methodological parameters of nine HBQs (*n* = 6).

Analyte	Linear Range (μg/L)	Linear	R^2^	Recovery, % (RSD, %)			LOD (ng/L)	LOQ (ng/L)
				Spiked at 10 ng/L	Spiked at 50 ng/L	Spiked at 500 ng/L		
2,5-DCBQ	4~1000	2980.143x − 7053.38	0.9994	109.3 (12.9)	115.3 (11.3)	102.3 (9.0)	0.5	0.15
2,5-DBBQ	4~1000	4369.877x − 5346.38	0.9987	86.0 (8.4)	79.4 (12.5)	94.1 (7.7)	0.5	1.5
2,6-DCBQ	4~1000	6618.669x − 7598.79	0.9989	123.3 (6.9)	101.2 (12.7)	109.5 (10.5)	0.1	0.3
2,6-DBBQ	4~1000	14,314.21x − 25,460.2	0.9993	84.0 (10.8)	75.2 (15.5)	96.3 (9.2)	0.2	0.6
TetraCBQ	4~1000	3656.618x − 3000.86	0.9989	112.5 (11.9)	126.6 (10.7)	105.3 (8.8)	0.7	2.1
TetraBBQ	4~1000	6012.050x − 5526.22	0.9987	87.0 (6.8)	80.5 (11.0)	107.6 (9.2)	0.3	0.9
TetraC-1,2-BQ	4~1000	1173.196x − 7213.38	0.9963	108.5 (14.7)	117.3 (9.2)	105.6 (11.5)	0.5	1.5
DBDMBQ	4~1000	337.1917x + 1170.10	0.9974	82.3 (7.8)	77.5 (14.3)	97.2 (12.1)	0.3	0.9
DCMBQ	4~1000	3991.976x − 6143.96	0.9974	73.5 (9.6)	80.6 (12.5)	91.9 (8.3)	0.1	0.3

**Table 3:** Comparison with other literature methods.

Quantitative Technique	Purification Method	Extraction Solvent/Column /Membrane	Sample Loading Time (min)	Analytes	Linear Range	LOD	Detected Concentration (ng/L)	Ref.
GC-ECD	LLE ^a^	Methyl-*tert*-butyl ether (M*t*BE)	-	2,6-DCBQ and 2,6-DBBQ	-	0.8–0.9 ng/L	2.4–20.5 ng/L	[19]
LC-MS	SPE ^b^	Waters Oasis HLB cartridges (6 mL, 200 mg)	62.5	4 HBQs	1–100 ng/L	0.3–2.0 ng/L	0.5–165 ng/L	[21]
LC-MS				12 HBQs	1–20 ng/L	0.2–6.6 ng/L	2.5–21.3 ng/L	[22]
LC-MS				4 HBQs and 4 OH-HBQs	-	0.03–0.8 ng/L	0.3–20.3 ng/L	[23]
LC-MS	MSPE ^c^	SDB-RPS membrane	10	9 HBQs	4–1000 ng/L	0.1–0.7 ng/L	2.0–5.1 ng/L	This work

- refers to relevant information not mentioned in the literature. ^a^ LLE refers to liquid–liquid extraction. ^b^ SPE refers to solid-phase extraction. ^c^ MSPE refers to membrane solid-phase extraction.

## Data Availability

Data will be made available on request.
